# Preparation of ZnNiAl-LDHs microspheres and their adsorption behavior and mechanism on U(VI)

**DOI:** 10.1038/s41598-021-01133-5

**Published:** 2021-11-03

**Authors:** Yanquan Ouyang, Yuanxin Xu, Limei Zhao, Mingzhan Deng, Pengfei Yang, Guowen Peng, Guojun Ke

**Affiliations:** 1 School of Chemistry and Chemical Engineering, University of South China, Hengyang, 421001 China; 2 Hunan Key Laboratory for the Design and Application of Actinide Complexes, University of South China, Hengyang, 421001 China; 3 China Nuclear Construction Key Laboratory of High Performance Concrete, University of South China, Hengyang, 421001 China; 4 Hunan Provincial Key Laboratory of High Performance Special Concrete, University of South China, Hengyang, 421001 China

**Keywords:** Chemical engineering, Environmental chemistry

## Abstract

Ternary zinc-nickel-aluminum hydrotalcites (ZnNiAl-LDHs) were prepared by hydrothermal synthesis. The structure and morphology of the materials were characterized using X-ray diffraction (XRD), fourier transform infrared spectroscopy (FT-IR), scanning electron microscopy (SEM), nitrogen adsorption–desorption (BET) and other test techniques. ZnNiAl-LDHs was applied in the treatment of uranium-containing wastewater, the effects of initial pH of the solution, adsorption temperature and contact time on its adsorption performance were systematically investigated, and the adsorption performance of ZnNiAl-LDHs and ZnAl-LDHs on uranyl ions were compared. The result showed that ZnNiAl-LDHs were 3D microspheres self-assembled from flakes, with a specific surface area of 102.02 m^2^/g, which was much larger than that of flake ZnAl-LDHs (18.49 m^2^/g), and the saturation adsorption capacity of ZnNiAl-LDHs for uranyl ions (278.26 mg/g) was much higher than that of ZnAl-LDHs for uranyl ions (189.16 mg/g), so the ternary ZnNiAl-LDHs had a more excellent adsorption capacity. In addition, kinetic and thermodynamic studies showed that the adsorption process of ZnNiAl-LDHs on uranyl ions conformed to the pseudo-second-order kinetic model and Langmuir isotherm model. The positive value of Δ*H* and the negative value of Δ*G* indicated that the adsorption process was endothermic and spontaneous. The adsorption mechanism was analyzed by X-ray energy spectroscopy (EDS), fourier transform infrared spectroscopy (FT-IR) and X-ray photoelectron spectroscopy (XPS). The results showed that the adsorption of uranyl ions by ZnNiAl-LDHs mainly consisted of complexation and ion substitution. The research results prove that ZnNiAl-LDHs is an adsorbent with low cost and excellent performance, and it has a good application prospect in the field of uranium-containing wastewater treatment.

## Introduction

In the context of the increasing depletion of traditional fossil energy sources, nuclear energy has been widely used as an economical, efficient and clean energy source^1,2^. The development of nuclear energy plays an important role in meeting the energy demand, but the large amount of uranium-containing wastewater generated during the production of the nuclear industry poses a series of problems^3–6^. In order to achieve the removal and enrichment of U(VI) from uranium-containing wastewater, many technologies have been developed, including chemical precipitation method^7^, ion exchange method^8^, membrane separation method^9^, solvent extraction method^10^ and adsorption method^11,12^, etc. Among various technologies, adsorption method has the advantages of low cost, environmental protection and simple operation. Therefore, it is considered to be the most promising technology for the treatment of uranium-containing wastewater.

Hydrotalcite is a kind of layered structure material composed of positively charged main laminates and negatively charged interlayer anions, also known as layered double hydroxides (LDHs), the general structural formula can be expressed as [M^2+^_1-x_M^3+^_x_(OH^−^)_2_]^x+^(A^n−^)_x/n_·mH_2_O, where M^2+^ is a divalent cation, such as Mg^2+^, Ni^2+^ or Zn^2+^; M^3+^ is a trivalent cation, such as Al^3+^ or Fe^3+^; A^n−^ is an anion, such as CO_3_^2−^, NO_3_^−^ and C1^−^, etc.^13–18^. Due to its high specific surface area, abundant oxygen-containing functional groups and interlayer ion exchange capacity, it can be widely used in wastewater treatment through modification treatment. Kameda et al.^19^ synthesized ZnAl-LDHs modified with carboxymethyl-β-cyclodextrin (CM-β-CD) ions to adsorb metal ions in aqueous solutions, and determined that Li^+^, Ni^2+^, Cu^2+^, Pb^2+^ and Nd^3+^ could be adsorbed by CM-β-CD-ZnAl-LDHs, and examined the selectivity of adsorption. Zhu et al.^20^ synthesized amino-trimethylene phosphonic acid (ATMP) intercalated ZnA1-LDHs by anion-exchange method and used it to absorb Cu^2+^ and Pb^2+^ from wastewater. The adsorption capacity of the modified LDHs for pollutants was significantly improved. Besides, there have been researches related to the use of hydrotalcite as adsorbent for uranium-containing wastewater treatment by researchers. For example, Xie et al.^21^ prepared binary flake FeAl-LDHs as adsorbent by ultrasonic-assisted precipitation method and used it for the removal of U(VI) in wastewater solutions. The experimental result showed that the maximum adsorption capacity of FeAl-LDHs was 113.64 mg/g at 308 K through the synergistic effect of adsorption-reduction. Li et al.^22^ synthesized binary flake CaAl-LDHs by modified coprecipitation method under ultrasonic technology. The result showed that the maximum adsorption capacity of CaAl-LDHs for U(VI) was 54.79 mg/g, and the maximum adsorption rate was 90.28%. However, the binary hydrotalcites prepared by the traditional method are mostly in the form of hexagonal flakes with low specific surface area, which limits the application of hydrotalcites in the field of adsorption. In recent years, in order to expand the application of layered hydrotalcite compounds, the preparation of ternary and multivariate hydrotalcites with special properties by introducing other divalent or trivalent metal ions on the basis of Mg–Al hydrotalcite is the focus of research in this field at home and abroad.

In this paper, ZnNiAl-LDHs with 3D microspherical structure were prepared by a simple hydrothermal synthesis method and applied in the treatment of uranium-containing wastewater. XRD, FT-IR, SEM, BET, EDS and XPS characterization methods were used to analyze the material and explain the adsorption mechanism. The effects of various factors on the adsorption performance of ZnNiAl-LDHs for uranyl ions were discussed. In order to understand the adsorption process deeply, the adsorption kinetic, thermodynamic and isotherm were analyzed. The results show that ternary hydrotalcites synthesized in this study has a good adsorption performance for uranyl ions.

## Experimental sections

### Materials and reagents

Zinc nitrate hexahydrate (Zn(NO_3_)_2_·6H_2_O), nickel nitrate hexahydrate (Ni(NO_3_)_2_·6H_2_O), aluminum nitrate nonahydrate (Al(NO_3_)_3_·9H_2_O) were purchased from Sinopharm Chemical Reagent Co., Ltd. Urea (CO(NH_2_)_2_), anhydrous sodium acetate (CH_3_COONa), chloroacetic acid (ClCH_2_COOH), arsenazo III, uranyl nitrate hexahydrate (UO_2_(NO_3_)_2_·6H_2_O) were purchased from Shanghai Macleans Biochemical Technology Co., Ltd. All chemicals (analytical grade) were used as received without any further purification. Deionized water was made by the laboratory.

### Preparation of ZnA1-LDHs and ZnNiA1-LDHs

0.010 mol Zn(NO_3_)_2_·6H_2_O, 0.005 mol Ni(NO_3_)_2_·6H_2_O, 0.005 mol A1(NO_3_)_3_·9H_2_O and 0.066 mol CO(NH_2_)_2_ were dissolved in 100 mL deionized water, stirred for 1 h at room temperature, and then transferred to 100 mL in a reaction kettle lined with polytetrafluoroethylene, put it in an oven at 100 °C to react for 24 h. After the reaction, the reactor was cooled to room temperature, and the precipitate was collected by centrifugation. The precipitate was washed with deionized water to neutrality, and then dried at 60 °C for 12 h to obtain ZnNiA1-LDHs.

0.015 mol Zn(NO_3_)_2_·6H_2_O, 0.005 mol A1(NO_3_)_3_·9H_2_O and 0.066 mol CO(NH_2_)_2_ were used as raw materials, ZnA1-LDHs were prepared using the same steps as ZnNiA1-LDHs.

### Characterization

Fourier transform infrared spectroscopy (Nicolet-iS10 type, Thermo Fisher Scientific, USA) was used for IR analysis of samples. The samples were characterized by XRD with an X-ray diffractometer (Bruker D8, Bruker, Germany), Cu target, Kα radiation, Ni filter, tube voltage 40 kV, tube current 40 mA, scanning range 2*θ* = 5° ~ 90°. The morphology of samples was characterized by scanning electron microscope (QUANTA FEG 450 type, FEI company, USA) and transmission electron microscope (Talos F200X type, Thermo Scientific company, USA). The specific surface area was analyzed and determined by Brunauer–Emmett–Teller (Micro for TriStar II Plus 2.02, Micro Company, USA). Energy dispersive spectroscopy was used to analyze the element content of samples. X-ray photoelectron spectroscopy (Escalab 250Xi, Thermo Fisher Scientific, USA) was used for XPS analysis to explore the adsorption mechanism.

### Adsorption experiments

Batch adsorption experiments under different experimental conditions such as pH, time and temperature were carried out in a 100 mL conical flask. Add 5.00 mg of adsorbent to a conical flask containing 40 mL of uranium solution and adjust pH with HNO_3_ or NaOH solution. The adsorption experiment was carried out on a reciprocating water bath shaking table, and the supernatant was taken for detection after the reaction. The concentration of U(VI) was determined by an UV spectrophotometer at a wavelength of 652 nm using arsenazo III spectrophotometry. In order to reduce the experimental error, all experimental data were the average of 3 repeated experiments. The equilibrium adsorption capacity (*q*_*e*_, mg/g) and adsorption rate (*R*, %) of LDHs for U(VI) were calculated by the following two equations:

1 $$q_{e} = \frac{{\left( {C_{0} - C_{e} } \right) \times V}}{m}$$

2 $$R = \frac{{\left( {\mathop C\nolimits_{0} - \mathop C\nolimits_{e} } \right)}}{{\mathop C\nolimits_{0} }} \times 100\%$$

where *C*_0_ and *C*_*e*_ were the initial concentration and equilibrium concentration (mg/L) of the uranium solution, *V* was the solution volume (L), and *m* was the mass of adsorbent (g).

## Results and discussion

### Characterization

**Figure 1:**
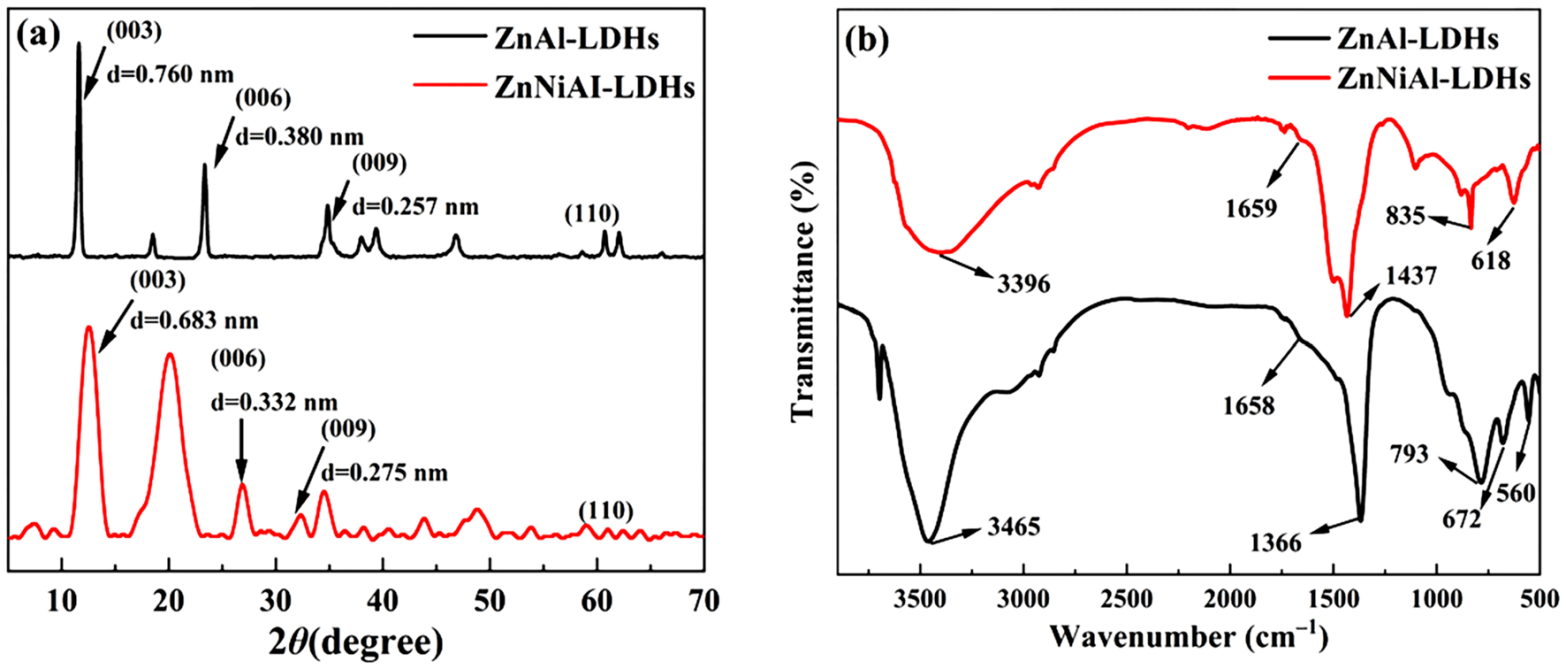
XRD patterns (**a**) and FT-IR patterns (**b**) of ZnAl-LDHs and ZnNiAI-LDHs.

Figure 1a shows the XRD patterns of ZnA1-LDHs and ZnNiA1-LDHs. As shown, ZnA1-LDHs and ZnNiA1-LDHs have typical characteristic peaks of LDHs at 2*θ* = 11.6°, 23.37°, 34.86°, 60.73° and 2*θ* = 12.95°, 26.88°, 32.29°, 61.08°, respectively (JCPDS No. 89-0460), and these characteristic peaks correspond to (003), (006), (009) and (110) crystal planes^23^. The symmetry and intensity of these peaks are relatively high, indicating that the two prepared LDHs have high crystallinity. Meanwhile, the d-values corresponding to the (003), (006) and (009) crystal plane diffraction peaks of ZnA1-LDHs and ZnNiA1-LDHs show a simple multiplicative relationship (0.760 nm = 2 × 0.380 nm ≈ 3 × 0.257 nm, 0.683 nm ≈ 2 × 0.332 nm ≈ 3 × 0.275 nm), implying that ZnAl-LDHs and ZnNiA1-LDHs have a typical layered structure^24^.

Figure 1b is the FT-IR spectrum of ZnA1-LDHs and ZnNiA1-LDHs, the bands at about 3400 cm^−1^ and 1650 cm^−1^ are the stretching and bending vibration peaks of –OH, respectively, and the peak near 1400 cm^−1^ is related to the asymmetric stretching vibration of interlayer CO_3_^2−^. In the low-frequency region, the peaks of 500–900 cm^−1^ can be regarded as metal–oxygen-metal and oxygen-metal–oxygen bending vibration peaks^25^. The above conclusions indicate that two LDHs materials were successfully prepared.

**Figure 2:**
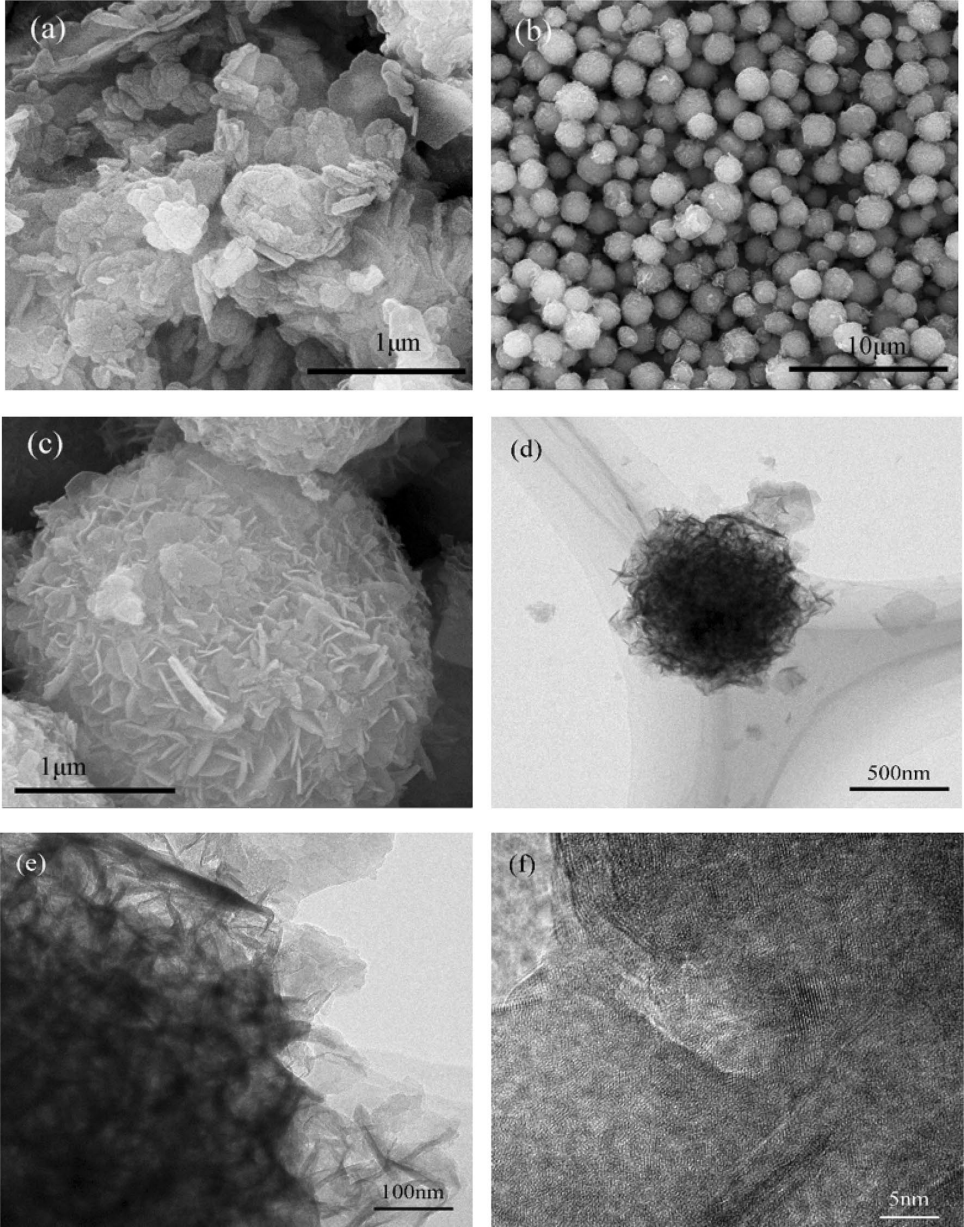
SEM images of ZnAl-LDHs (**a**) and ZnNiAI-LDHs (**b**,**c**). TEM images of ZnNiAl-LDHs (**d**–**f**).

Figure 2a is the SEM picture of ZnAl-LDHs. It can be seen from the picture that ZnAl-LDHs present a regular hexagonal sheet structure with the size of 0.3–0.5 μm, and the sheet layer is relatively thin, which is similar to the general LDHs structure^26^. Figure 2b,c are SEM pictures of ZnNiAl-LDHs. The pictures show that ZnNiAl-LDHs are composed of a large number of uniform, well-dispersed microspheres with an average diameter of 1–2 μm. Figure 2d,e are TEM images of a single microsphere of ZnNiAl-LDHs, which are consistent with the SEM image observed in Fig. 2c. The microsphere is formed by nanosheets growing from the center to the spherical surface. At the same time, the lattice fringes of the material can be clearly observed through the high magnification transmission electron microscope image of Fig. 2f. The above characterization results indicate that the prepared ZnNiAl-LDHs crystal structure is complete and the crystallinity is excellent, which is consistent with the XRD characterization.

**Figure 3:**
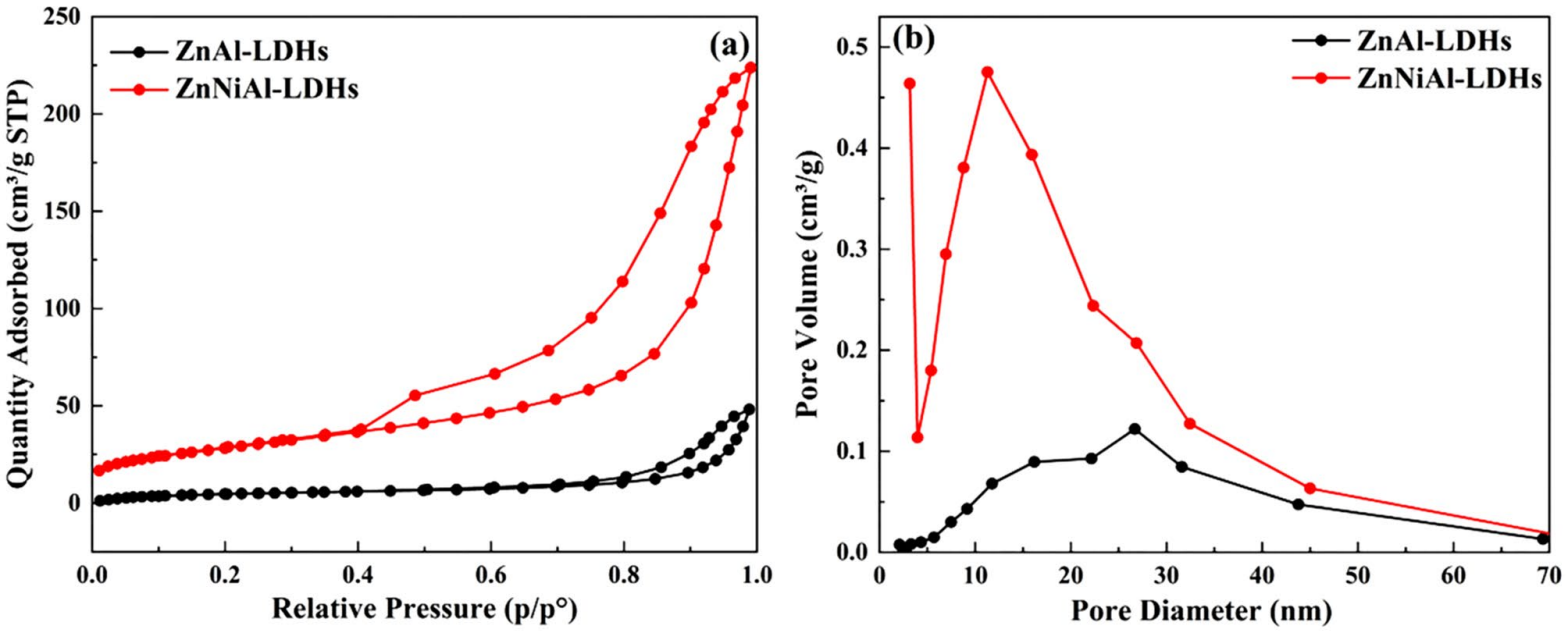
Nitrogen adsorption–desorption isotherms (**a**) and pore size distributions (**b**) of ZnAl-LDHs and ZnNiAl-LDHs.

Figure 3a is the nitrogen adsorption–desorption isotherms of ZnAl-LDHs and ZnNiAl-LDHs. The structure of two materials are similar, and the adsorption and desorption isotherms are typical type IV isotherms, which belong to mesoporous adsorption materials^27^. The hysteresis loops of two materials both appear in the higher relative pressure range, which are of type H_3_, indicating that there are slit holes formed by the accumulation of flake particles, so the two materials may be flake or layered materials similar to clay^28^. As can be seen from the pore size distributions diagram in Fig. 3b, the pore size distributions of ZnAl-LDHs and ZnNiAl-LDHs are relatively wide, ranging from 5 to 45 nm and 5 to 25 nm, respectively. Therefore, ZnNiAl-LDHs has a more uniform pore structure. In addition, the BET characterization parameters are shown in Table 1, the specific surface area, pore volume and pore diameter of ZnNiAl-LDHs are 102.02 m^2^/g, 0.346 cm^3^/g and 13.573 nm, respectively. While the specific surface area, pore volume and pore size of ZnAl-LDHs are 18.49 m^2^/g, 0.075 cm^3^/g and 16.115 nm, respectively, suggesting that the microsphere structure of ZnNiAl-LDHs has richer spatial structure and can provide more binding sites than the flake structure, which is beneficial to show its excellent adsorption performance.

**Table 1:** The BET characterization results of ZnAl-LDHs and ZnNiAl-LDHs.

Samples	Surface area (m^2^/g)		Pore volume (cm^3^/g)	Pore size (nm)	
ZnAl-LDHs	18.49		0.075	16.115	
ZnNiAl-LDHs	102.02		0.346	13.573	

### Effect of initial pH

**Figure 4:**
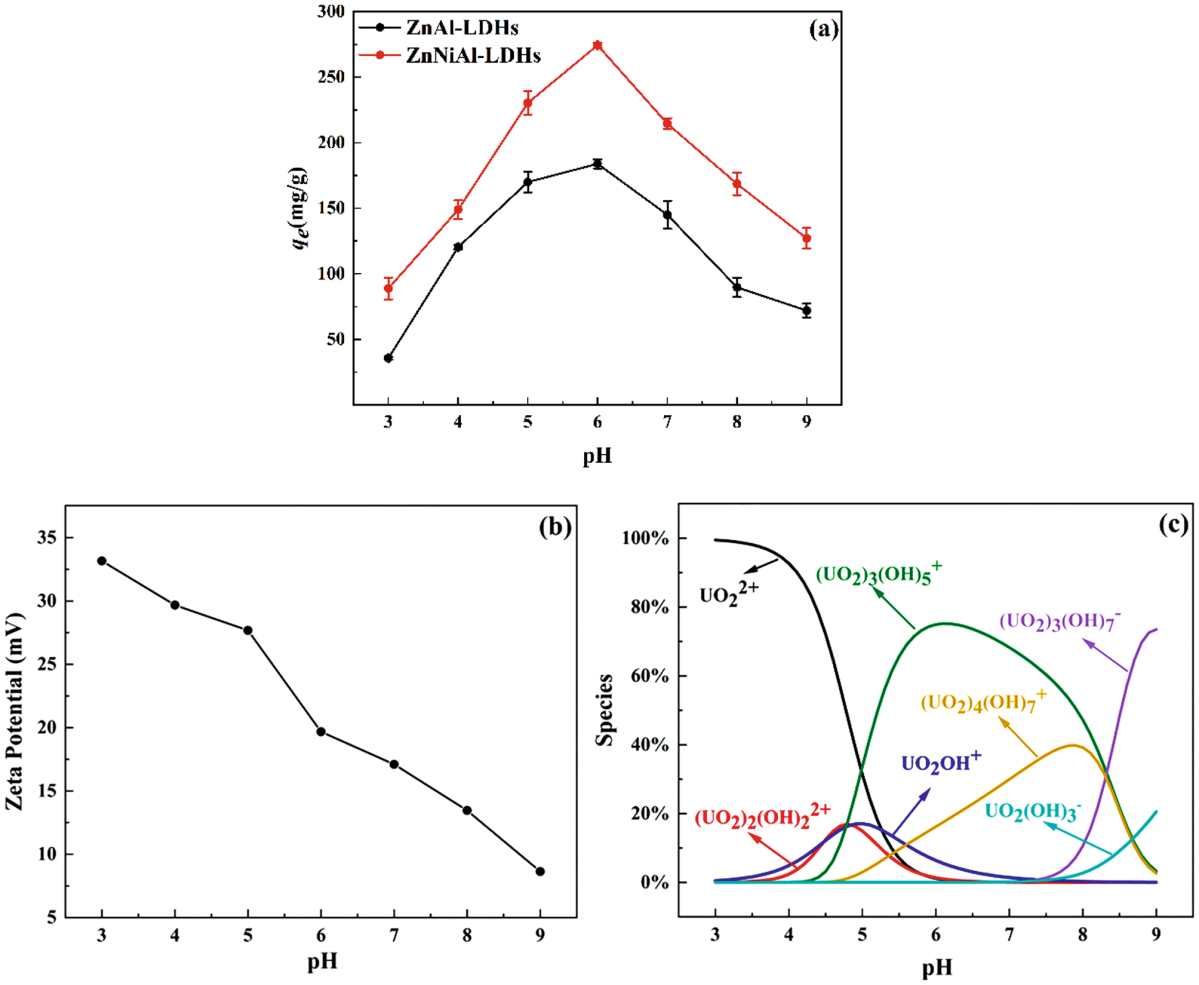
Effect of pH on the adsorption of U(VI) by ZnAl-LDHs and ZnNiAl-LDHs (**a**), Zeta potential of ZnNiAl-LDHs (**b**) and the distribution of U(VI) species in aqueous solution as a function of pH (**c**) (*C*_0_ (U) = 35 mg L^−1^,* m/V* = 0.125 g L^−1^,* t* = 8 h (ZnAl-LDHs) and 6 h (ZnNiAl-LDHs),* T* = 303 K.

In the adsorption of U(VI) by LDHs, the initial pH in the solution is an important influencing factor, so the adsorption of U(VI) on LDHs under different pH conditions were carefully investigated in this experiment. Figure 4a shows that ZnAl-LDHs and ZnNiAl-LDHs have similar adsorption trends in the pH range of 3 to 9. Therefore, ZnNiAl-LDHs were selected for Zeta potential analysis to investigate the relationship between the species distribution of uranium in solution at different pH and the surface charge properties of the adsorbent, and the Zeta potential of ZnNiAl-LDHs and U(VI) species distribution with pH are shown in Fig. 4b,c.

When the pH is 3 ~ 4, due to the high buffering capacity of LDHs, adsorbent will partially dissolve and release OH^−^ to increase the pH. At the same time, H^+^ will also compete with U(VI) for adsorption^29^, so the adsorption capacity is reduced. With the increase of pH, the removal efficiency of U(VI) increases rapidly. The adsorption capacities of ZnAl-LDHs and ZnNiAl-LDHs both reach the peak value at pH 6, and then gradually decrease. The main reason for this phenomenon may be that at pH < 6, the uranium in solution exists mainly as UO_2_^2+^, UO_2_OH^+^ and (UO_2_)_3_(OH)_5_^+^. As the pH increases, the deprotonation of LDHs increases, and the positive charge on the surface is relatively less, and the repulsion of the adsorbent from U(VI) decreases, so the adsorption capacity gradually increases^30^. When the pH is greater than 6, U(VI) mainly exists in the form of negative complexes such as (UO_2_)_3_(OH)_7_^−^ and UO_2_(OH)_3_^−^ and carbonate complexes such as UO_2_(CO_3_)_2_^2−^ and UO_2_(CO_3_)_3_^4−^^31^. These compounds can exist stably in the solution and are difficult to be adsorbed by adsorbents, resulting in the decrease of adsorption capacities, suggesting that the adsorption process is mainly controlled by the complexation reaction of U(VI) with surface functional groups rather than electrostatic forces. Therefore, in subsequent experiments, ZnAl-LDHs and ZnNiAl-LDHs were adsorbed at pH 6. On the whole, the adsorption performance of ZnNiAl-LDHs is better than that of ZnAl-LDHs. The reason is that ZnNiAl-LDHs has a larger specific surface area, so it has better pH buffering capacity and adsorption capacity.

### Effect of contact time and adsorption kinetics

Contact time is a key factor affecting the mass transfer rate of U(VI) during adsorption. In order to determine the adsorption equilibrium time and perform adsorption kinetic analysis, an investigation of the effect of contact time on adsorption performance was conducted.

**Figure 5:**
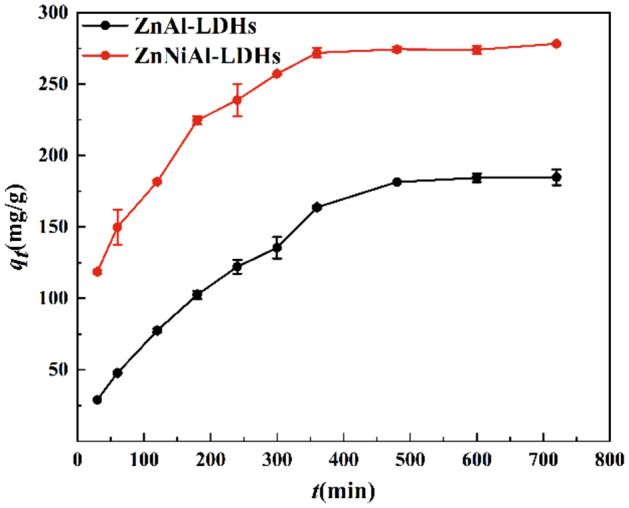
Adsorption kinetics of U(VI) on ZnAl-LDHs and ZnNiAl-LDHs (*C*_0_ (U) = 35 mg L^−1^,* m/V* = 0.125 g L^−1^, pH = 6.0,* T* = 303 K).

It can be seen from Fig. 5 that in the first 2 h, the adsorption of uranyl ions by ZnAl-LDHs and ZnNiAl-LDHs increase rapidly. This phenomenon is due to the fact that in the initial stage of adsorption, the surface of LDHs provides a large number of active sites to combine with U(VI)^32^. As the reaction time increases, the available binding sites gradually decrease. U(VI) needs to slowly diffuse to the inner surface, causing the adsorption rate to gradually slow down. And finally the adsorption capacities of ZnAl-LDHs and ZnNiAl-LDHs for uranyl ions reach the adsorption equilibrium at 8 h and 6 h, respectively. Since the adsorption capacity of ZnNiAl-LDHs was much larger than that of ZnAl-LDHs, ZnNiAl-LDHs was chosen as the object of study in the subsequent experiments to investigate the effects of various factors on the adsorption performance of LDHs for uranyl ions and to probe the mechanism of the interaction between LDHs and U(VI). In order to describe the relationship between adsorption capacity and contact time and to analyze the adsorption mechanism, the pseudo-first-order kinetic model, the pseudo-second-order kinetic model and Elovich model are used to fit the adsorption process of LDHs on U(VI), and the three models are expressed as follows:

pseudo-first-order kinetic model:

3 $$\ln \left( {q_{e} - q_{t} } \right) = \ln q_{e} - k_{1} t$$

pseudo-second-order kinetic model:

4 $$\frac{t}{{q_{t} }} = \frac{1}{{k_{2} q_{e}^{2} }} + \frac{t}{{q_{e} }}$$

Elovich model:

5 $$q_{t} = \frac{1}{\beta }\ln \left( {\alpha \beta } \right) + \frac{1}{\beta }\ln \left( t \right)$$

where *k*_1_ (min^−1^) and *k*_2_ (g/(mg·min)) are the rate constants of two models, *α* (mg/g × min) and *β* (mg/g) are the Elovich model constants, *q*_*t*_ (mg/g) is the adsorption capacity at time t and *q*_*e*_ (mg/g) is the adsorption capacity at equilibrium.

**Figure 6:**
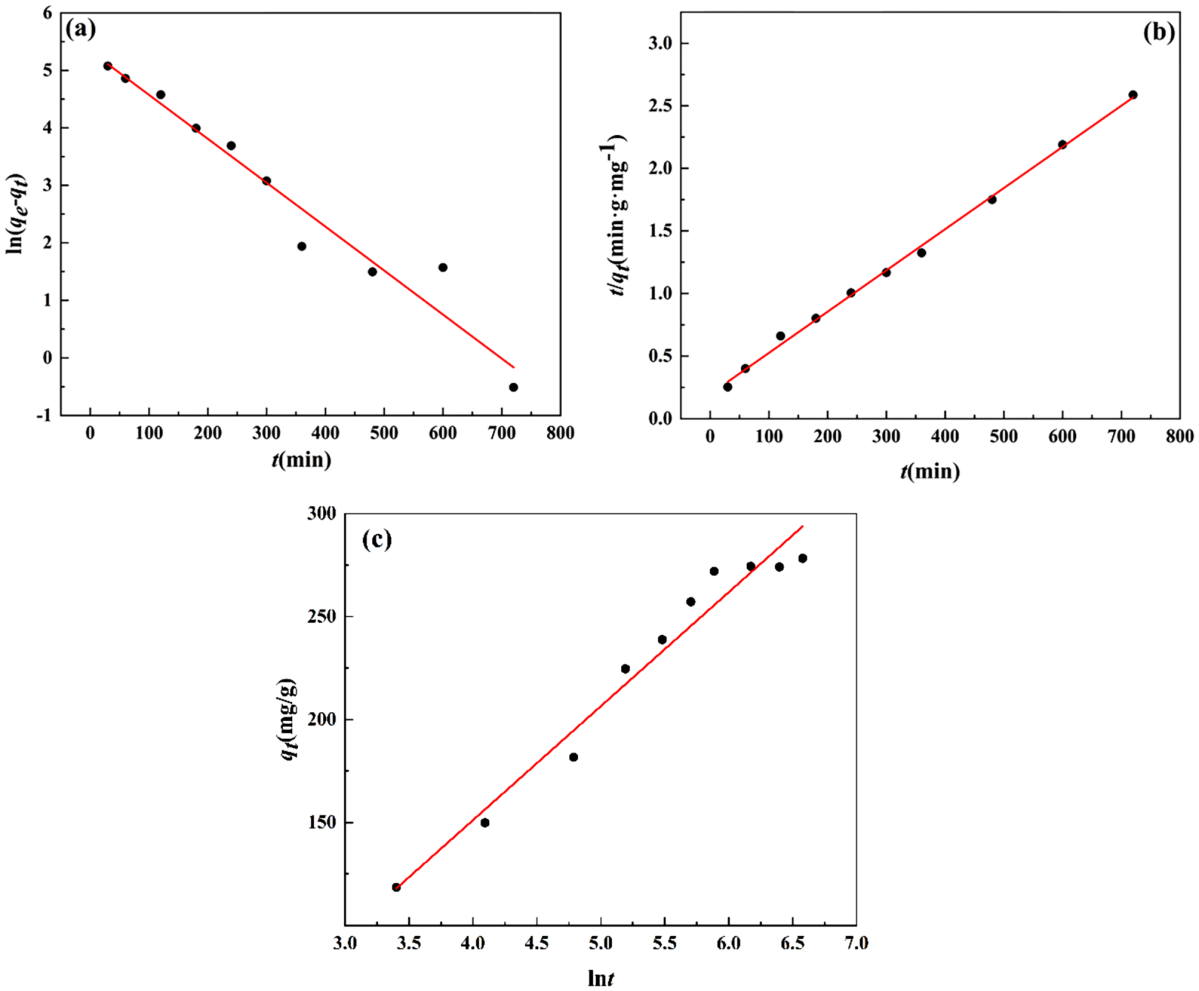
Pseudo-first-order kinetic model (**a**), pseudo-second-order kinetic model (**b**) and Elovich model (**c**) of U(VI) adsorption by ZnNiAl-LDHs.

Figure 6 show the ftting curves of the pseudo-first-order kinetic model (a), pseudo-second-order kinetic model (b) and Elovich model (c), the related parameters are listed in Table 2. The R^2^ value of the pseudo-second-order kinetic model is higher than those of the pseudo-first-order kinetic model and Elovich model, moreover, the theoretical equilibrium adsorption capacity (303.95 mg/g) of ZnNiAl-LDHs calculated by the pseudo-second-order kinetic model is closer to the real experimental result (278.26 mg/g), implying that the pseudo-second-order kinetic model can better describe the kinetic process of adsorption of uranium ions by ZnNiAl-LDHs, indicating the adsorption process of the adsorbent is dominated by chemisorption on a homogeneous surface^33,34^.

**Table 2:** The adsorption kinetic parameters of the adsorption of uranyl ions by ZnNiAl-LDHs.

Pseudo-first-order model			Pseudo-second-order model			Elovich model		
*q*_*e*_	*k*_1_	R^2^	*q*_*e*_	*k*_2_	R^2^	*α*	*β*	R^2^
208.26	0.00764	0.950	303.95	5.52 × 10^–5^	0.997	15.537	0.018	0.960

The units of *q*_*e*_ (mg/g), *k*_1_ (min^−1^), *k*_2_ (g/(mg·min)), *α* (mg/g × min), *β* (mg/g).

### Adsorption isotherms

**Figure 7:**
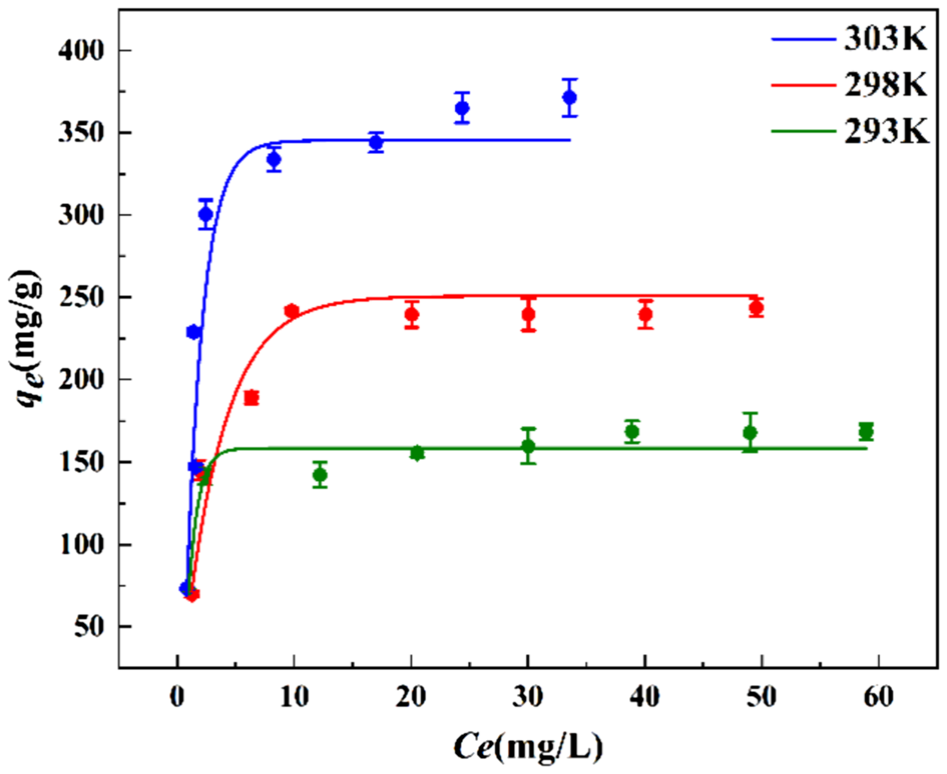
Adsorption isotherms of U(VI) on ZnNiAl-LDHs (*m/V* = 0.125 g L^−1^, pH = 6.0,* t* = 6 h).

The adsorption isotherm is a curve describing the change of adsorption capacity with equilibrium concentration at a certain temperature. In order to further evaluate the adsorption capacity of ZnNiAl-LDHs for U(VI), the adsorption isotherms of the initial concentration of uranium solution in the range of 10 ~ 80 mg/L were studied at 293 K, 298 K and 303 K, respectively. It can be seen from the Fig. 7 that in the initial stage, as the concentration increases, the adsorption capacity of ZnNiAl-LDHs on U(VI) increases rapidly. This is because under the force of the concentration gradient, the adsorbent can more fully contact U(VI), the adsorption sites on the surface are easier to bind to U(VI). However, when the initial concentration continues to increase, the adsorption capacity of ZnNiAl-LDHs on U(VI) tends to balance, the reason is that adsorption site of the materials is almost completely occupied, and the adsorption reaches saturation^35^. Langmuir model, Freundlich model and Temkin model were used to fit the adsorption isotherms, the three models are expressed as:

Langmuir model:

6 $$\frac{{C_{e} }}{{q_{e} }} = \frac{1}{{K_{L} q_{m} }} + \frac{{C_{e} }}{{q_{m} }}$$

Freundlich model:

7 $$\ln q_{e} = \ln K_{F} + \frac{1}{n}\ln C_{e}$$

Temkin model:

8 $$q_{e} = \frac{{{\text{R}}T}}{{b_{T} }}\ln A_{T} + \frac{{{\text{R}}T}}{{b_{T} }}\ln C_{e}$$

where *q*_*e*_ (mg/g) is the adsorption capacity of ZnNiAl-LDHs in adsorption equilibrium, *q*_*m*_ (mg/g) is the theoretical maximum adsorption capacity, *k*_*L*_ (L/mg) is the adsorption constant of Langmuir model, *k*_*F*_ ((mg/g) (L·mg)^1/n^) and n are the adsorption constants related to the adsorption capacity and adsorption strength of Freundlich model, respectively. *A*_T_ (L/mg) and *b*_T_ (J/mol) are Temkin model constants.

**Figure 8:**
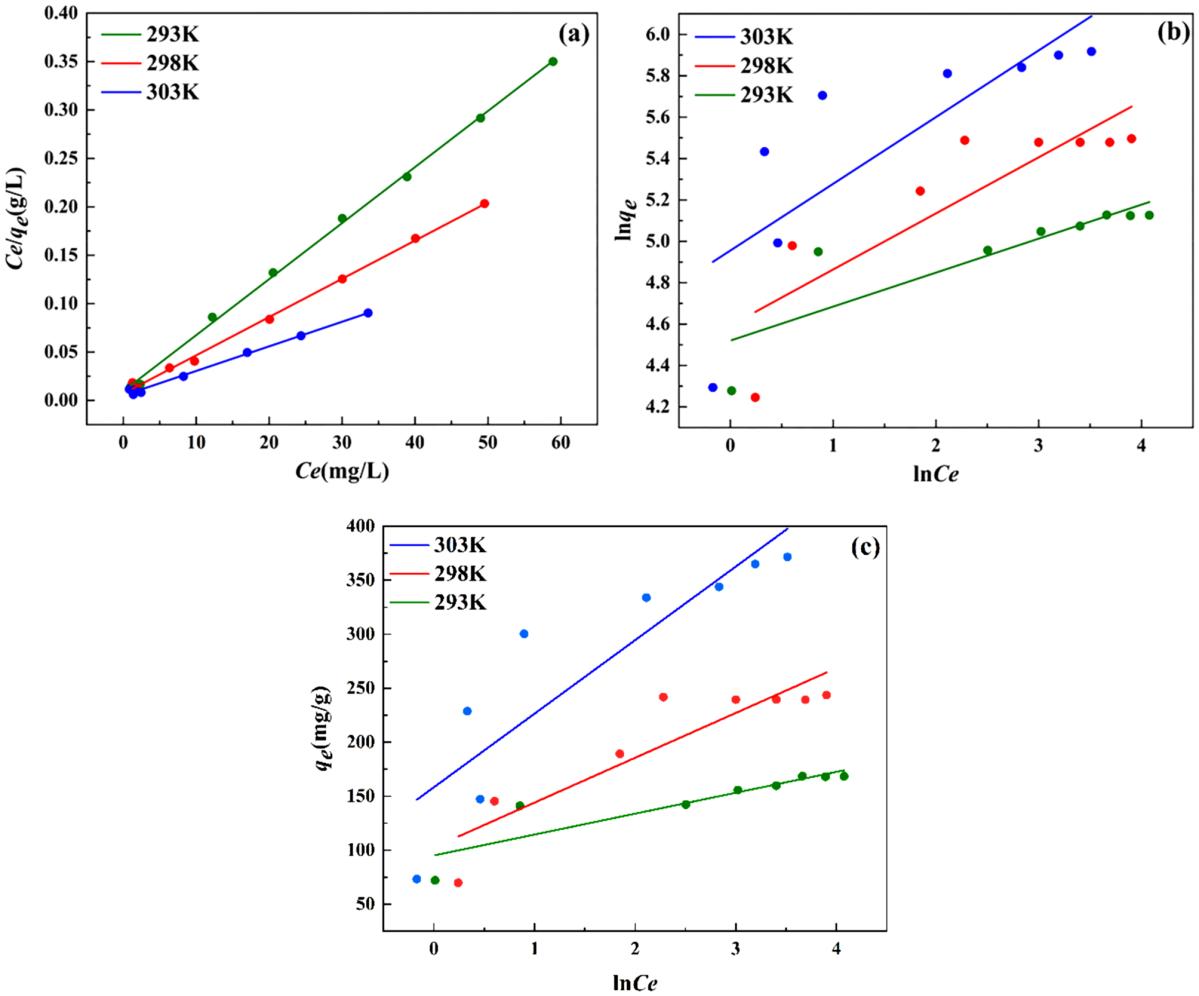
Langmuir model (**a**), Freundlich model (**b**) and Temkin model (**c**) of ZnNiAl-LDHs adsorption of U(VI).

The ftting results of Langmuir model, Freundlich model and Temkin model are shown in Fig. 8, and the parameters fitted by three models are shown in Table 3, the Langmuir model (R^2^ > 0.99) can better describe the adsorption data, indicating that adsorption process is a homogeneous surface adsorption process dominated by monolayer adsorption^36^. In addition, under the same other conditions, the adsorption capacity of ZnNiAl-LDHs increases significantly with the increase of temperature, which illustrates that the adsorption of U(VI) by ZnNiAl-LDHs is an endothermic reaction. Therefore, a proper temperature rise is conducive to the progress of adsorption.

**Table 3:** The adsorption isotherm parameters of the adsorption of uranyl ions by ZnNiAl-LDHs.

T (K)	Langmuir Isotherm			Freundlich Isotherm			Temkin model		
	*q*_*m*_	*k*_*L*_	R^2^	*k*_*F*_	n	R^2^	*A*_T_	*b*_T_	R^2^
293	172.71	0.607	0.999	91.878	6.087	0.692	136.067	125.826	0.765
298	253.16	0.558	0.998	98.804	3.690	0.687	11.824	59.671	0.790
303	393.70	0.501	0.993	141.98	3.102	0.596	10.200	36.969	0.755

The units of *q*_*m*_ (mg/g), *k*_*L*_ (L/mg), *k*_*F*_ ((mg/g)(L·mg)^1/n^), *A*_T_ (L/mg), *b*_T_ (J/mol).

### Adsorption mechanism

**Figure 9:**
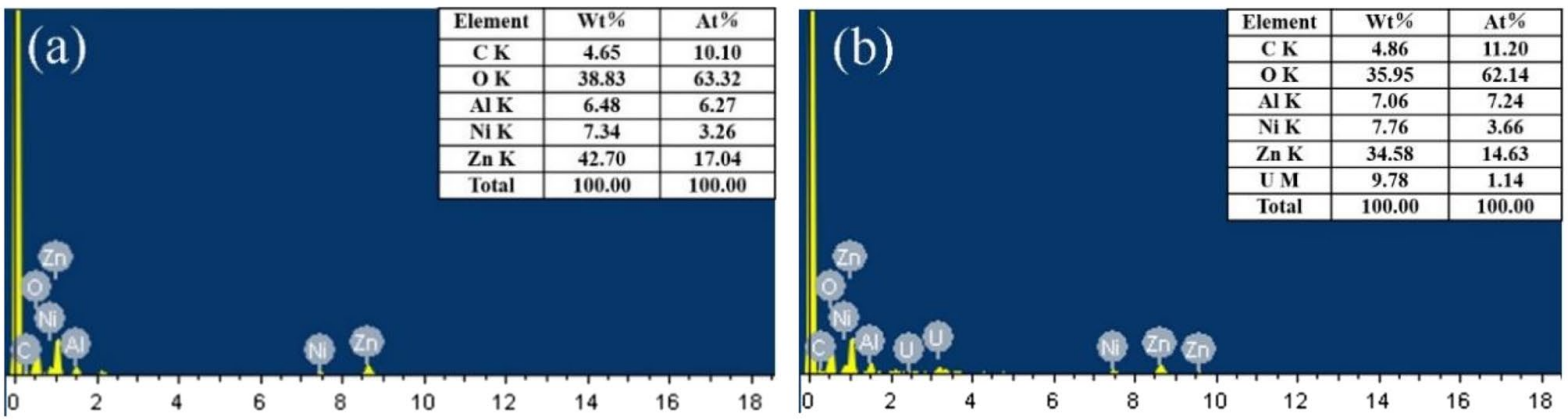
EDS patterns of ZnNiAl-LDHs before (**a**) and after (**b**) U(VI) adsorption.

**Figure 10:**
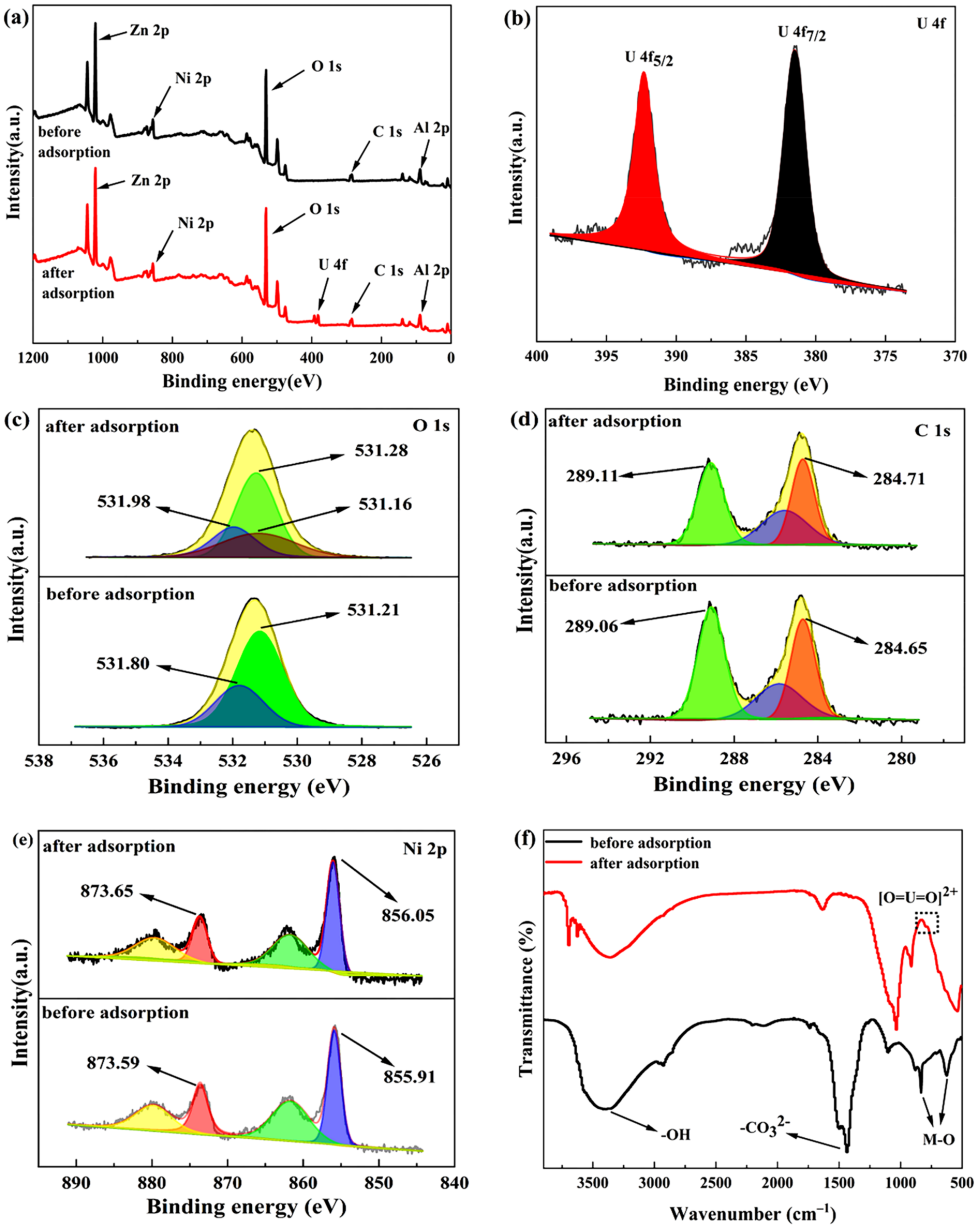
Wide scan XPS spectra (**a**), high-resolution XPS spectra of U 4f (**b**), O 1s (**c**), C 1s (**d**), Ni 2p (**e**) and FT-IR spectra (**f**) of ZnNiAl-LDHs before and after adsorption of U(VI).

In order to explore the adsorption mechanism of ZnNiAl-LDHs on U(VI), EDS, FT-IR and XPS were analyzed. Figure 9 shows the EDS patterns of ZnNiA1-LDHs before and after the adsorption of U(VI). Compared with Fig. 9a, the content of U appears in Fig. 9b, confirming that ZnNiA1-LDHs has an adsorption effect on U(VI). Figure 10a is XPS full scan spectrum before and after ZnNiA1-LDHs adsorption of U(VI), the results show that material surface contains C, O, Zn, Ni and Al elements, and the adsorbed material shows the characteristic peaks of U(VI), indicating that the material has adsorbed U(VI). The U 4f spectrum in Fig. 10b shows two characteristic peaks at 381.5 eV and 392.3 eV, which correspond to U 4f_7/2_ and U 4f_5/2_, respectively^37^. In addition, the O 1s and C 1s of ZnNiA1-LDHs were subjected to peak separation processing, and the results were shown in Fig. 10c,d. The O 1s spectrum before adsorption can be divided into two parts, M–O (metal containing oxygen bond) and –OH at 531.21 eV and 531.80 eV, respectively^38^. Compared with the O 1s spectrum before adsorption, the peaks after adsorption move to higher binding energy and the occupied peak area is reduced, confirming that M–O and -OH in ZnNiA1-LDHs have reacted with U(VI). In addition, the O 1 s spectrum after adsorption shows a new peak at 531.16 eV, which may be due to the formation of U–O^39^. Similarly, the C 1 s spectrum shows HCO_3_^−^ and CO_3_^2−^ related groups at 284.65 eV and 289.06 eV, and about 285.0 eV corresponds to the C elemental calibration energy peak^40^. The changes in the positions of above two peaks and the weakening of the peak intensities indicate that HCO_3_^−^ and CO_3_^2−^ between the ZnNiA1-LDHs layers are chelated with U(VI)^41^. In addition, according to Fig. 10e, it is found that the structural element of adsorbed ZnNiA1-LDHs may have changed. Ni 2p peaks are observed at 855.91 eV (Ni 2p_3/2_) and 873.59 eV (Ni 2p_1/2_), and the small peaks are related satellite peaks^42,43^. The positions and peak intensities of Ni 2p peaks have changed after adsorption, indicating the changes in the local bonding environment and chemical structure. The reason may be that Ni^2+^ has the same valence state and similar ionic radius as UO_2_^2+^, so UO_2_^2+^ may have ion exchange with the nickel oxygen bond on the surface of ZnNiA1-LDHs^44,45^.

**Figure 11:**
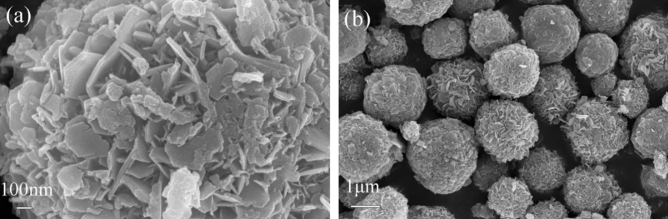
SEM images of ZnNiAl-LDHs before (**a**) and after (**b**) U(VI) adsorption.

Figure 10f shows the FT-IR spectra of ZnNiAl-LDHs before and after adsorption. The spectrum shows that the adsorbed ZnNiA1-LDHs still have the characteristic peaks of LDHs, and the characteristic peak of UO_2_^2+^ appears at about 830 cm^−1^^46^, indicating that the material successfully adsorbs U(VI). In addition, the peak intensities of the characteristic peaks corresponding to –OH, –CO_3_^2−^ and M–O are all weakened and the peak positions are shifted, which may be due to the reaction with U(VI). The SEM images of ZnNiAl-LDHs after adsorption are shown in Fig. 11a,b. The material still maintains a good microsphere structure, but a few irregular nanosheets appear on the surface, probably due to the reaction of the constituents of LDHs with U(VI), which causes some structural damage. Combined with FT-IR and SEM analysis, it can be seen that ZnNiAl-LDHs still maintain their original structure after adsorption, meaning that the material has great stability.

Based on the above analysis, this study proposes that the potential mechanism for removing U(VI) may be the complexation of U(VI) with surface hydroxyl groups, chelation with interlayer functional ligands and ion substitution.

### Effect of temperature and thermodynamic data

**Figure 12:**
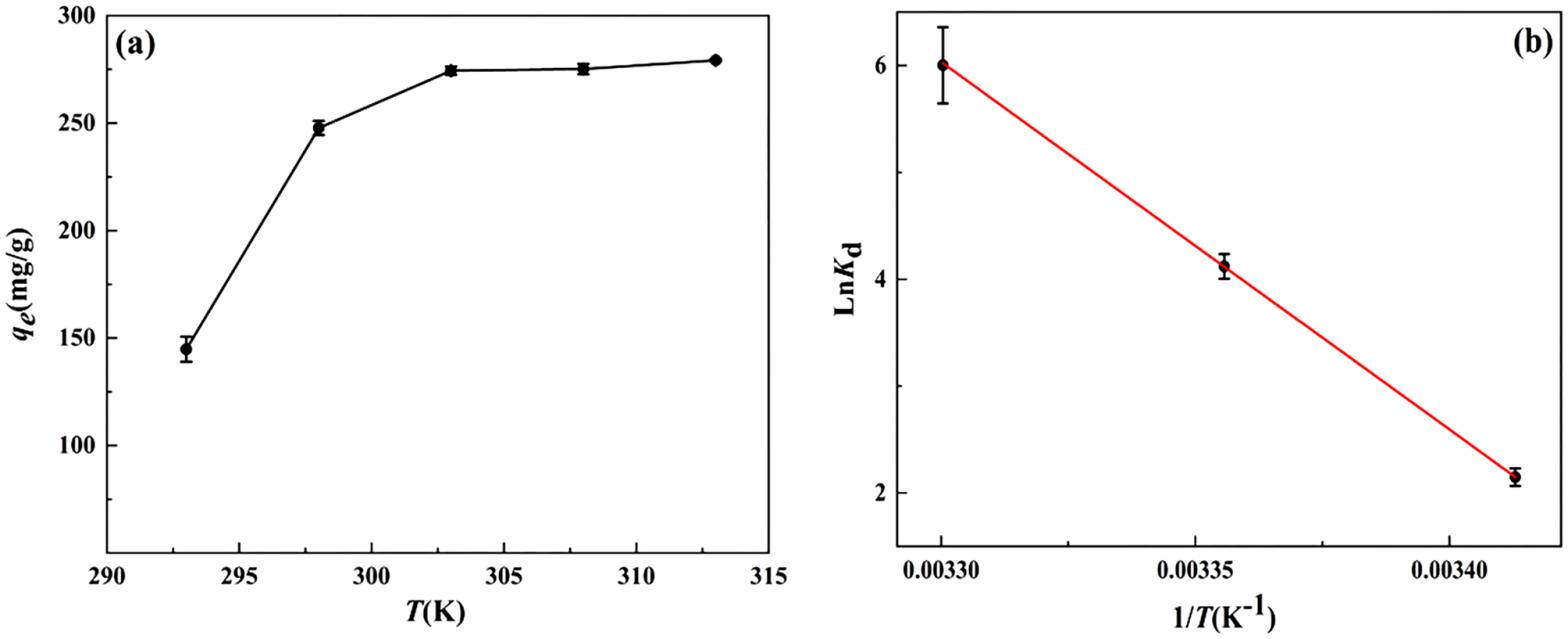
Effect of temperature (**a**) and the ln*K*_d_-1/*T* diagram of ZnNiAl-LDHs on the adsorption of U(VI) (**b**) (*C*_0_ (U) = 35 mg L^−1^,* m/V* = 0.125 g L^−1^, pH = 6.0,* t* = 6 h).

In order to discuss the adsorption performance of ZnNiAl-LDHs for U(VI) from the perspective of thermodynamics, different adsorption temperatures were selected for research to determine the type of adsorption reaction (endothermic or exothermic). As shown in Fig. 12, when *T* < 303 K, with the increase of temperature, the adsorption capacity shows an upward trend. The main reason is that the increase in temperature aggravates the movement of molecules and promotes the contact of U(VI) with active sites on the surface of ZnNiAl-LDHs^47^. When *T* = 303 K, the adsorption capacity reaches the maximum, and the U(VI) in original solution is almost completely adsorbed. When *T* > 303 K, as the temperature increases, adsorption capacity basically keeps the peak value, so 303 K is selected as the optimal adsorption temperature. Thermodynamic parameters such as enthalpy (Δ*H*), entropy (Δ*S*) and Gibbs free energy (Δ*G*) are used to describe the process. These parameters can be obtained according to the following equations:

9 $$\ln K_{d} = \frac{{\Delta H^{0} }}{{{\text{R}}T}} + \frac{{\Delta S^{0} }}{{\text{R}}}$$

10 $$\Delta G^{0} = \Delta H^{0} - T\Delta S^{0}$$

where R (8.314 J/(mol·K)) is the ideal gas constant, *T* (K) is the reaction temperature, and *K*_d_ is the solid–liquid partition coefficient; the calculated slope and intercept of the curve of ln*K*_d_ versus 1/*T* correspond to ∆*H*^0^/R and ∆*S*^0^/R, respectively. The Δ*G*^0^ value is calculated by the Eq. (10), and the thermodynamic parameters are shown in the Table 4:

**Table 4:** Adsorption thermodynamic parameters of the adsorption of uranyl ions by ZnNiAl-LDHs.

*T* (K)	ln*K*_d_	Δ*G*^0^ (kJ/mol)	*∆H*^0^ (kJ/mol)	*∆S*^0^ (J/(mol K))	R^2^
293	2.14753	− 5.2339	285.832	993.4	0.999
298	4.12125	− 10.201			
303	6.00164	− 15.168			

According to the data in Table 4, the positive value of ∆*H*^0^ indicates that the adsorption process is endothermic, and this result is also consistent with the analysis of adsorption isotherms. The negative value of Δ*G*^0^ means that the adsorption of U(VI) on ZnNiA1-LDHs is spontaneous^48^. In addition, the positive value of ∆*S*^0^ reveals that the adsorption process is proceeding in the direction of increasing disorder^49^. In this study, the potential adsorption mechanism of ZnNiA1-LDHs to remove U(VI) in the solution is ion substitution, the material structure changes, which leads to more chaos in the entire system, this analysis is also in line with the previous analysis results.

### Effect of cations

**Figure 13:**
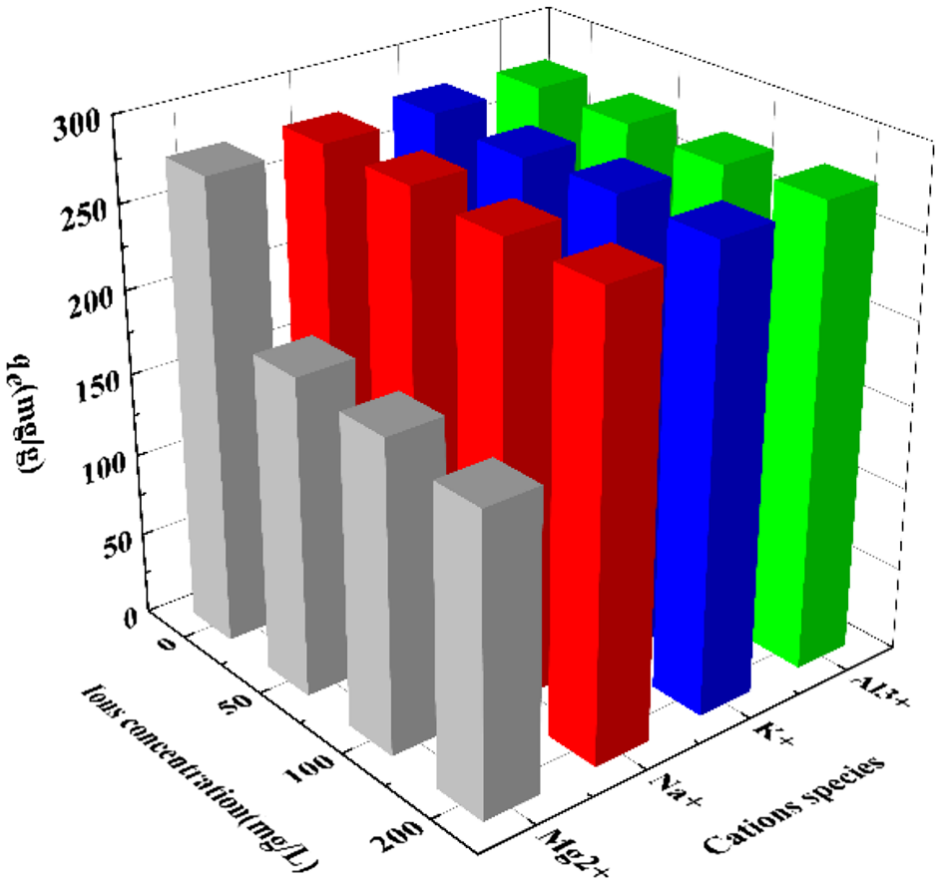
Effect of cations on the adsorption of U(VI) by ZnNiA1-LDHs (*C*_0_ (U) = 35 mg L^−1^,* m/V* = 0.125 g L^−1^, pH = 6.0,* t* = 6 h,* T* = 303 K).

In the presence of cations such as Na^+^, K^+^, Mg^2+^ and Al^3+^, the adsorption capacity of ZnNiA1-LDHs for U(VI) in the solution is shown in Fig. 13. The result shows that in most cases, ZnNiA1-LDHs has excellent selectivity to UO_2_^2+^, which is attributed to the fact that Na^+^, K^+^ and Al^3+^ have different valence states and ion radii compared with UO_2_^2+^, but Mg^2+^ has the same valence state and similar ionic radius as Ni^2+^ and UO_2_^2+^. According to the literature, the main laminate of LDHs is formed by the co-edge of octahedra of divalent and trivalent metal ions, so the structural stability of the minimum structural motif MO_6_ octahedra may determine the stability of the main laminate, and the introduction of Mg^2+^ into the LDHs materials can obtain a stable laminate structure. Therefore, Mg^2+^ may partly exchange with the nickel-oxygen bonds on the surface of ZnNiA1-LDHs, thereby being introduced into the hydrotalcite structure, occupying adsorption sites, affecting the adsorption of UO_2_^2+^ in the solution by the adsorbent, resulting in a decrease in uranium adsorption^50,51^.

### Reusability of adsorbent

**Figure 14:**
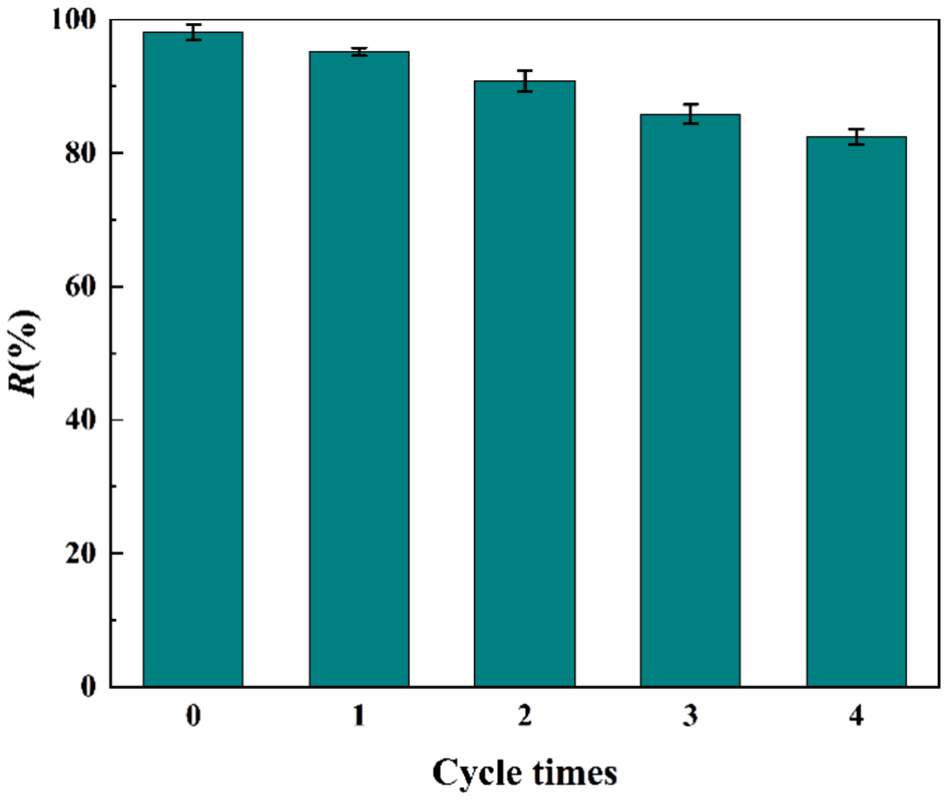
Effect of cycle times on adsorption of U(VI) by ZnNiA1-LDHs (*C*_0_ (U) = 35 mg L^−1^,* m/V* = 0.125 g L^−1^, pH = 6.0,* t* = 6 h,* T* = 303 K).

In practical applications, the regenerability of adsorbents is an important basis for evaluating materials. 0.1 mol/L Na_2_CO_3_ solution was selected as the desorption agent, mixed with the adsorbed ZnNiA1-LDHs and shaken for 12 h, washed with deionized water to neutrality, and the dried material was subjected to repeated adsorption experiments. As shown in Fig. 14, after 4 cycles, the removal rate of U(VI) by ZnNiA1-LDHs still maintained more than 80%, proving that the material has good regeneration performance and recyclability.

## Conclusion

ZnNiAl-LDHs with microspherical structure were prepared by a simple hydrothermal synthesis method, and the microspheres had uniform particle size and good dispersibility. The adsorption properties of ZnNiAl-LDHs and ZnAl-LDHs for U(VI) were studied, experimental results showed that ZnNiAl-LDHs exhibited better adsorption capacity for U(VI) due to the larger specific surface area and more active sites in the microsphere structure of ZnNiAl-LDHs. The adsorption of ZnNiAl-LDHs on U(VI) reached equilibrium within 6 h, and the fit of experimental data was consistent with the pseudo-second-order kinetic model, while the Langmuir isotherm model fitted the adsorption isotherm well, indicating that the adsorption process was dominated by monolayer coverage and chemosorption. The thermodynamic parameters indicated that the adsorption of U(VI) by ZnNiAl-LDHs was an endothermic and spontaneous process. In addition, the presence of common co-existing cations in the solution had no obvious effect on the removal of U(VI) in most cases, indicating that the adsorbent had good selectivity for U(VI). Moreover, ZnNiAl-LDHs could still exhibit good adsorption performance after several cycles. Finally, the adsorption mechanism was explained by FT-IR and XPS characterization. The result showed that the adsorption of U(VI) on ZnNiAl-LDHs involved complexation and ion exchange. Therefore, ZnNiAl-LDHs can be used as an effective low-cost adsorbent for the treatment of uranium-containing wastewater.

## References

[1] Zeng M (2016). Review of nuclear power development in China: Environment analysis, historical stages, development status, problems and countermeasures. Renew. Sustain. Energy Rev..

[2] Aviel V, Erik L, Sanne L (2014). Assessment of the actual sustainability of nuclear fission power. Renew. Sustain. Energy Rev..

[3] Lili C (2017). Efficient sorption and reduction of U(VI) on zero-valent iron-polyaniline-graphene aerogel ternary composite. J. Colloid Interface Sci..

[4] Zou Y (2016). Controllable synthesis of Ca-Mg-Al layered double hydroxides and calcined layered double oxides for the efficient removal of U(VI) from wastewater solutions. ACS Sustain. Chem. Eng..

[5] Bajwa BS, Kumar S, Singh S, Sahoo SK, Tripathi RM (2019). Uranium and other heavy toxic elements distribution in the drinking water samples of SW-Punjab, India. J. Radiat. Res. Appl. Sci..

[6] Arnaud M (2018). An evidence of chemically and physically mediated migration of 238U and its daughter isotopes in the vicinity of a former uranium mine. J. Environ. Radioact..

[7] Sayali K, Anand B, Shree Kumar A (2013). Bioprecipitation of uranium from alkaline waste solutions using recombinant Deinococcus radiodurans. J. Hazard. Mater..

[8] Chayan B (2014). Nano-cerium vanadate: A novel inorganic ion exchanger for removal of americium and uranium from simulated aqueous nuclear waste. J. Hazard. Mater..

[9] Chen Z (2014). Uranium removal and microbial community in a H_2_-based membrane biofilm reactor. Water Res..

[10] Yuan L (2014). Solvent extraction of U(VI) by trioctylphosphine oxide using a room-temperature ionic liquid. Science China Chem..

[11] Xingjun W (2019). Surface hydroxylation of SBA-15 via alkaline for efficient amidoxime-functionalization and enhanced uranium adsorption. Sep. Purif. Technol..

[12] de Decker J, de Clercq J, Vermeir P, van der Voort P (2016). Functionalized metal-organic-framework CMPO@MIL-101(Cr) as a stable and selective rare earth adsorbent. J. Mater. Sci..

[13] Xu Y, Ke G, Yin J, Lei W, Yang P (2018). Synthesis of thiol-functionalized hydrotalcite and its application for adsorption of uranium (VI). J. Radioanal. Nucl. Chem..

[14] Seddighi H, Khodadadi Darban A, Khanchi A, Fasihi J, Koleini J (2017). LDH(Mg/Al:2)@montmorillonite nanocomposite as a novel anion-exchanger to adsorb uranyl ion from carbonate-containing solutions. J. Radioanal. Nucl. Chem..

[15] Zou Y (2016). Coagulation behavior of graphene oxide on nanocrystallined Mg/Al layered double hydroxides: Batch experimental and theoretical calculation study. Environ. Sci. Technol..

[16] Yu S (2017). Layered double hydroxide intercalated with aromatic acid anions for the efficient capture of aniline from aqueous solution. J. Hazard Mater..

[17] Yao W (2017). Enhanced removal of methyl orange on calcined glycerol-modified nanocrystallined Mg/Al layered double hydroxides. Chem. Eng. J..

[18] Zhou Y (2020). Simultaneous removal of cationic and anionic heavy metal contaminants from electroplating effluent by hydrotalcite adsorbent with disulfide (S^2-^) intercalation. J. Hazard Mater..

[19] Kameda T, Takaizumi M, Kumagai S, Saito Y, Yoshioka T (2020). Adsorption of various metals by carboxymethyl-β-cyclodextrin-modified Zn Al layered double hydroxides. Appl. Clay Sci..

[20] Zhu S, Asim Khan M, Wang F, Bano Z, Xia M (2020). Rapid removal of toxic metals Cu^2+^ and Pb^2+^ by amino trimethylene phosphonic acid intercalated layered double hydroxide: A combined experimental and DFT study. Chem. Eng. J..

[21] Xie L (2017). Sono-assisted preparation of Fe(II)-Al(III) layered double hydroxides and their application for removing uranium (VI). Chem. Eng. J..

[22] Li Y (2013). Ultrasound assisted synthesis of Ca–Al hydrotalcite for U (VI) and Cr (VI) adsorption. Chem. Eng. J..

[23] Liu J (2017). A novel luminescence probe based on layered double hydroxides loaded with quantum dots for simultaneous detection of heavy metal ions in water. J. Mater. Chem. C..

[24] Wang Q, Feng Y, Feng J, Li D (2011). Enhanced thermal- and photo-stability of acid yellow 17 by incorporation into layered double hydroxides. J. Solid State Chem..

[25] Wang J, Kang D, Yu X, Ge M, Chen Y (2015). Synthesis and characterization of Mg–Fe–La trimetal composite as an adsorbent for fluoride removal. Chem. Eng. J..

[26] Zheng Y-M, Li N, Zhang W-D (2012). Preparation of nanostructured microspheres of Zn–Mg–Al layered double hydroxides with high adsorption property. Colloids Surf. A.

[27] Jiexin L (2019). Preparation of sulfhydryl functionalized magnetic SBA-15 and its high-efficiency adsorption on uranyl ion in solution. Environ. Sci. Pollut. Res. Int..

[28] Zhang F, Song Y, Song S, Zhang R, Hou W (2015). Synthesis of magnetite-graphene oxide-layered double hydroxide composites and applications for the removal of Pb(II) and 2,4-dichlorophenoxyacetic acid from aqueous solutions. ACS Appl Mater Interfaces..

[29] Tan L (2015). Enhanced adsorption of uranium (VI) using a three-dimensional layered double hydroxide/graphene hybrid material. Chem. Eng. J..

[30] Liu Y, Yang P, Li Q, Liu Y, Yin J (2019). Preparation of FeS@Fe_3_O_4_ core–shell magnetic nanoparticles and their application in uranyl ions removal from aqueous solution. J. Radioanal. Nucl. Chem..

[31] Cheng W, Wan T, Wang X, Wu W, Hu B (2018). Plasma-grafted polyamine/hydrotalcite as high efficient adsorbents for retention of uranium (VI) from aqueous solutions. Chem. Eng. J..

[32] Shakur HR, Rezaee Ibrahim Saraee K, Abdi MR, Azimi G (2016). Selective removal of uranium ions from contaminated waters using modified-X nanozeolite. Appl. Radiat. Isot..

[33] Chen H (2018). Enhanced adsorption of U(VI) and (241)Am(III) from wastewater using Ca/Al layered double hydroxide@carbon nanotube composites. J. Hazard Mater..

[34] Yang P (2019). Preparation of modified pomelo peel's pulp adsorbent and its adsorption to uranyl ions. R. Soc. Open Sci..

[35] Lv Z (2019). Enhanced removal of uranium(VI) from aqueous solution by a novel Mg-MOF-74-derived porous MgO/carbon adsorbent. J. Colloid Interface Sci..

[36] Fang C, Tao Q, Dai Y (2020). Amidoximated orange peel as a specific uranium scavenger. J. Radioanal. Nucl. Chem..

[37] Liu P (2020). Removal of U(VI) from aqueous solution using AO-artificial zeolite. J. Radioanal. Nucl. Chem..

[38] Deng L, Shi Z, Wang L, Zhou S (2017). Fabrication of a novel NiFe_2_O_4_/Zn-Al layered double hydroxide intercalated with EDTA composite and its adsorption behavior for Cr(VI) from aqueous solution. J. Phys. Chem. Solids.

[39] Yang D (2017). Rational design and synthesis of monodispersed hierarchical SiO_2_@layered double hydroxide nanocomposites for efficient removal of pollutants from aqueous solution. Chem. Eng. J..

[40] Calderon VS, Cavaleiro A, Carvalho S (2015). Chemical and structural characterization of ZrCNAg coatings: XPS, XRD and Raman spectroscopy. Appl. Surf. Sci..

[41] Lyu F (2019). Efficient and fast removal of Pb^2+^ and Cd^2+^ from an aqueous solution using a chitosan/Mg-Al-layered double hydroxide nanocomposite. J. Colloid Interface Sci..

[42] Song S (2018). Interaction of U(VI) with ternary layered double hydroxides by combined batch experiments and spectroscopy study. Chem. Eng. J..

[43] Lei C (2017). Superb adsorption capacity of hierarchical calcined Ni/Mg/Al layered double hydroxides for Congo red and Cr(VI) ions. J. Hazard Mater..

[44] Sun M (2015). High uptake of Cu^2+^, Zn^2+^ or Ni^2+^ on calcined MgAl hydroxides from aqueous solutions: Changing adsorbent structures. Chem. Eng. J..

[45] Liang X (2013). Sorption of metal cations on layered double hydroxides. Colloids Surf. A.

[46] Wang H (2012). Selective solid-phase extraction of uranium by salicylideneimine-functionalized hydrothermal carbon. J. Hazard Mater..

[47] Pengfei Y, Yuanxin X, Na Y, Yong A (2020). Preparation of uniform highly dispersed Mg–Al-LDHs and their adsorption performance for chloride ions. Ind. Eng. Chem. Res..

[48] Yin N, Ai Y, Xu Y, Ouyang Y, Yang P (2020). Preparation of magnetic biomass-carbon aerogel and its application for adsorption of uranium(VI). J. Radioanal. Nucl. Chem..

[49] Zhou H, Jiang Z, Wei S (2018). A new hydrotalcite-like absorbent FeMnMg-LDH and its adsorption capacity for Pb^2+^ ions in water. Appl. Clay Sci..

[50] Hudcová B, Veselská V, Filip J, Číhalová S, Komárek M (2018). Highly effective Zn(II) and Pb(II) removal from aqueous solutions using Mg-Fe layered double hydroxides: Comprehensive adsorption modeling coupled with solid state analyses. J. Clean. Prod..

[51] Yan H (2008). Theoretical study of the hexahydrated metal cations for the understanding of their template effects in the construction of layered double hydroxides. J. Mol. Struct..

